# Predatory beetles feed more pest beetles at rising temperature

**DOI:** 10.1186/s12898-016-0076-x

**Published:** 2016-04-15

**Authors:** Thomas Frank, Martin Bramböck

**Affiliations:** Department of Integrative Biology and Biodiversity Research, Institute of Zoology, University of Natural Resources and Life Sciences Vienna (BOKU), Gregor Mendel Straße 33, 1180 Vienna, Austria

**Keywords:** Biological pest control, Biomass intake, Carabid beetles, Climate warming, Feeding activity, Killing rate, *Meligethes aeneus*, Pollen beetle, Predator-pest interaction, Temperature increase

## Abstract

**Background:**

Climate warming is a challenge for many plants and animals as they have to respond to rising temperature. Rising temperature was observed to affect herbivores and predators. Activity-density of abundant predatory carabid beetles, which are considered important natural control agents of agricultural pests, was observed to increase at rising temperature. The pollen beetle *Meligethesaeneus* is one of the most important insect pests in European oilseed rape fields, and its larvae were observed to be important prey to carabid beetles. Therefore, we performed a laboratory experiment to detect whether rising temperature affects the number of pollen beetle larvae killed by five abundant carabids, and larval biomass ingested by carabids. In three climate chambers actual temperature (T1) was compared to temperatures increased by 3 °C (T2) and 5 °C (T3). This is the first study investigating the feeding of carabid predators on an arable pest insect spanning a realistic forecasted climate warming scenario of 3 and 5 °C, thus providing basic knowledge on that neglected research area. We hypothesized that carabids kill more pollen beetle larvae at rising temperature, and biomass intake by carabids increases with rising temperature.

**Results:**

Both beetle species and temperature had significant effects on the number of killed *Meligethes* larvae and larval biomass ingested by carabids. *Amara ovata*, *Harpalus distinguendus* and *Poecilus cupreus* killed significantly more pollen beetle larvae at T2 and T3 compared to T1. *Anchomenus dorsalis* killed significantly more larvae at T2 than T1, and *Harpalus affinis* showed no significant differences among temperatures. Biomass intake by *A. ovata*, *H. distinguendus* and *A. dorsalis* was significantly larger at T2 and T3 compared to T1. Biomass intake by *H. affinis* and *P. cupreus* did not significantly differ among temperatures. Among the five carabids tested *P. cupreus* exhibited the highest values for both number of killed larvae and biomass intake.

**Conclusions:**

Our lab results suggest a clear potential for higher feeding of pollen beetle larvae by carabid beetles at rising temperature. As rising temperature leads to increased activity of abundant arable carabids in the field, it may be expected that there is enhanced pest suppression under warmer field conditions.

**Electronic supplementary material:**

The online version of this article (doi:10.1186/s12898-016-0076-x) contains supplementary material, which is available to authorized users.

## Background

Despite controversy on whether increased concentrations of atmospheric greenhouse gas lead to rising global temperature [1], there is an increase in annual-mean global temperature of 0.78 °C since the beginning of the 20th century [2]. Irrespective of contradictory opinions there will be regions with elevated temperature and the present study, which covers scenarios with elevated temperatures, is designed for regions that undergo temperature increases, e.g. Austria [3].

Where climate warming takes place it is a challenge for many plants and animals as they have to respond to rising temperature [4]. Rising temperature was observed to affect herbivores [5, 6] and predators [7]. In particular *Meligethes viridescens*, an economically important pest beetle of oilseed rape, was modelled to increase in abundance at rising temperature [8], and activity-density of abundant predatory carabid beetles was observed to increase in an artificially warmed wheat field [9]. Carabid beetles fed more slugs and seeds at higher temperature [7, 10]. Overall, temperature was observed to be the key climatic variable increasing carabid catches by pitfall traps [11]. This means that activity-density of carabid beetles increases at rising temperature, which enhances encounter probability between carabids and epigeic pest individuals, which in turn may increase the beneficial impact of carabid beetles under warmer field conditions.

Many carabid beetles are polyphagous predators. They are commonly found in arable crops and considered important natural control agents of agricultural pests [12–16]. The carabid beetles *Anchomenus dorsalis* and *Poecilus cupreus* were observed to reduce populations of oilseed rape insect pests emerging within winter rape fields, when studied in field exclosure experiments [17]. Moreover, larval abundance of the major oilseed rape insect pests, *Meligethes aeneus* and *Ceutorhynchus napi*, was crucial in explaining the condition and density of the carabids *Amara similata* and *P. cupreus* under field conditions [18], which indicates that these pest insects are an important prey to carabid beetles. Oilseed rape is one of the most commonly used crops in Europe and its importance as a source for industrial and nutritional oil has been increasing worldwide during the last decades [19]. The pollen beetle *M.aeneus* is one of the most important insect pests in European oilseed rape fields [20, 21]. Damage caused by pollen beetles usually results in podless peduncles. The present laboratory study focuses on pest-predator interactions involving larval pollen beetles and five medium to medium-large carabid beetles abundantly found in Central European arable crops in spring, thus being potential major antagonists of oilseed rape pests. Because pollen beetles pupate in the soil they are potentially susceptible to epigeic carabids in the larval developmental stage or as pupae.

The specific aims of the present study were to determine whether rising temperature affects (1) the number of *M.aeneus* larvae killed by carabids, and (2) *M.aeneus* larval biomass ingested by carabids. Even though feeding of predatory carabid beetles upon pest insects has generally been explored under laboratory conditions [22], published works including different temperature regimes span a large range of temperatures of more than 15 °C. The present study is to our knowledge the first one investigating the feeding of carabid predators on an arable pest insect spanning a realistic forecasted climate warming scenario of 3 and 5 °C. We hypothesized that (1) carabids kill more *M.aeneus* larvae at rising temperature, and (2) biomass intake by carabids increases with rising temperature.

## Methods

### Carabid and pollen beetle sampling

The experiment was performed with the spring breeding carabid beetles *Amara ovata* (Fabricius), *Anchomenus dorsalis* (Ponttopidan), *Harpalus affinis* (Schrank), *H. distinguendus* (Duftschmid) and *Poecilus cupreus* (Linnaeus), and the nitidulid pollen beetle *Meligethes aeneus* (Fabricius). Adult carabid beetles and larval pollen beetles were collected in an oilseed rape field in Raasdorf Lower Austria (16°59´E, 48°23´N), East of Vienna. Beetle sampling and field access were orally arranged with the owner of the field. For carabid sampling 120 pitfall traps (plastic cups, diameter 70 mm, depth 70 mm) were installed on 6 May and emptied on 9, 14 and 17 May 2013. Carabid beetles were brought to the laboratory, kept in 18 × 13 × 4.5 cm plastic boxes at 10 °C and fed with fish food (trademark "Tetra“©) prior to the experiment. On 1, 9, 14 and 21 May 2013 flowering inflorescences of oilseed rape plants were brought to the laboratory and pollen beetle larvae were picked out of the flowers. Prior to the experiment pollen beetle larvae were kept in 20 × 8 × 8 cm plastic boxes at 10 °C and fed with rape pollen.

### Experiment

The experiment was conducted in late May to June 2013 in three climate chambers at three different temperature regimes for each chamber. The basis for the temperatures used in the present work were the mean temperatures in April and May 1961–1990 for the region where beetles were collected (data provided by the Central Institution for Meteorology and Geodynamics Austria, ZAMG). Mean basis temperature (T1) was 11.5 °C, which was continuously increased by 5 °C during the day and continuously decreased by 5 °C during the night, i.e. T1 ranged from 6.5 °C at night to 16.5 °C during the day time, with the mean temperature of 11.5 °C. Mean temperatures of temperature 2 (T2) and temperature 3 (T3) were increased by 3 °C (T2) and 5 °C (T3) with the appropriate continuous alterations of 5 °C during day and night. T2 ranged from 9.5 °C at night to 19.5 °C during the day time, with the mean temperature of 14.5 °C, and T3 ranged from 11.5 °C at night to 21.5 °C during the day time, with the mean temperature of 16.5 °C.

In all three chambers a photoperiod of 14 h light and 10 h dark was used imitating light conditions for that time when pollen beetle larvae occur in the field. The used scenarios of future rising temperatures of 3 and 5 °C are in the range of climate models for Eastern Austria that compare the period 1961–1990 with 2071–2100 [23].

Before the experiment was started carabids were divided into sexes and only females were used for the experiment because for some species we did not collect enough males. Prior to the experiment female beetles were weighed assuring that weight of carabid beetle species tested did not differ significantly among temperature regimes (*A. ovata*, T1 35.90 ± 1.27, T2 38.70 ± 0.50, T3: 39.20 ± 1.57, N = 18, P = 0.145; *A. dorsalis*, T1 13.97 ± 0.49, T2 14.66 ± 0.49, T3 15.58 ± 0.91, N = 30, P = 0.243; *H. affinis*, T1 66.01 ± 2.66, T2 62.76 ± 2.29, T3 64.68 ± 2.05, N = 36, P = 0.256; *H. distinguendus,* T1 57.63 ± 3.15, T2 61.40 ± 3.07, T3 60.77 ± 2.66, N = 36, P = 0.805; *P. cupreus*, T1 99.83 ± 4.72, T2 91.58 ± 3.60, T3 96.02 ± 3.31, N = 36, P = 0.309; mean weight ± SE in mg, ANOVA). Twelve replicates per temperature regime were used, summing up to 36 Petri dishes for *H. affinis, H. distinguendus and P. cupreus*. Six replicates per temperature regime were used for *A. ovata*, and ten for *A. dorsalis*, depending on carabid availability. Female beetles were placed individually in Petri dishes (diameter 60 mm, height 15 mm) and starved for 48 h. To keep humidity in the Petri dishes stable at 100 %, each dish was covered with a 50 mm diameter paper towel which was irrigated with 1 mL water. To prevent the towels from desiccation they were irrigated daily. After 48 h of carabid starvation pollen beetle larvae, which served as food for the carabids, were counted and weighed. Thereafter, they were put in the Petri dishes where they stayed for 72 h. After 72 h, the number of non-killed pollen beetle larvae was counted and the whole remaining larval biomass was weighed enabling to measure both number of killed larvae and biomass intake by carabids over 72 h. For three carabid species ad libitum food availability during 72 h was guaranteed by initially feeding 30 pollen beetle larvae (*A. dorsalis*) or 40 pollen beetle larvae (*A. ovata*, *H. distinguendus*), respectively. These numbers of prey items were based on feeding tests that have been performed for each carabid species in advance under the same conditions as the experiment. Also, *H. affinis* were offered 40 pollen beetle larvae per Petri dish. As two individuals fed more than expected, ten additional pollen beetle larvae were offered to these two individuals after 48 h. Referring to pre-feeding tests with *P. cupreus*, 60 pollen beetle larvae were offered per Petri dish. However, as nine individuals fed more than expected additional pollen beetle larvae were offered to them after 48 h (six times ten additional larvae, and once 15, 20 and 30 larvae, respectively), providing ad libitum food availability during 72 h.

### Statistical analyses

Effects of temperature (three temperature regimes) and beetle species (five carabid species) on the number of killed pollen beetle larvae and biomass intake by carabids were analysed with generalized linear models (GLM). The model for killed larvae was analyzed using Poisson distribution. Kruskal–Wallis tests were calculated for killed larvae for single carabid species to test for differences between temperatures, followed by the Nemenyi post hoc test for multiple comparisons. One-way ANOVAs were calculated for biomass intake for single carabid species to test for differences between temperatures, followed by the Tukey post hoc test for multiple comparisons. We checked the data for normality using the Kolmogorov–Smirnov test and diagnostic graphs, and for homogeneity of variances using the Levene test, and found them adequate, thus analyses were performed with untransformed data. As the carabid beetles used vary in size and body mass, we additionally calculated the number of killed pollen beetle larvae mg^−1^ and intake of larval pollen beetle biomass mg^−1^ among the five carabid species for all three temperatures together. As these data were not normally distributed one-way ANOVAs followed by the Tukey post hoc test were calculated with box-cox transformed data. Analyses were run with SPSS 21.0 (SPSS Inc., Chicago, IL, USA), and level of significance was defined at* P* < 0.05.

## Results

**Fig. 1:**
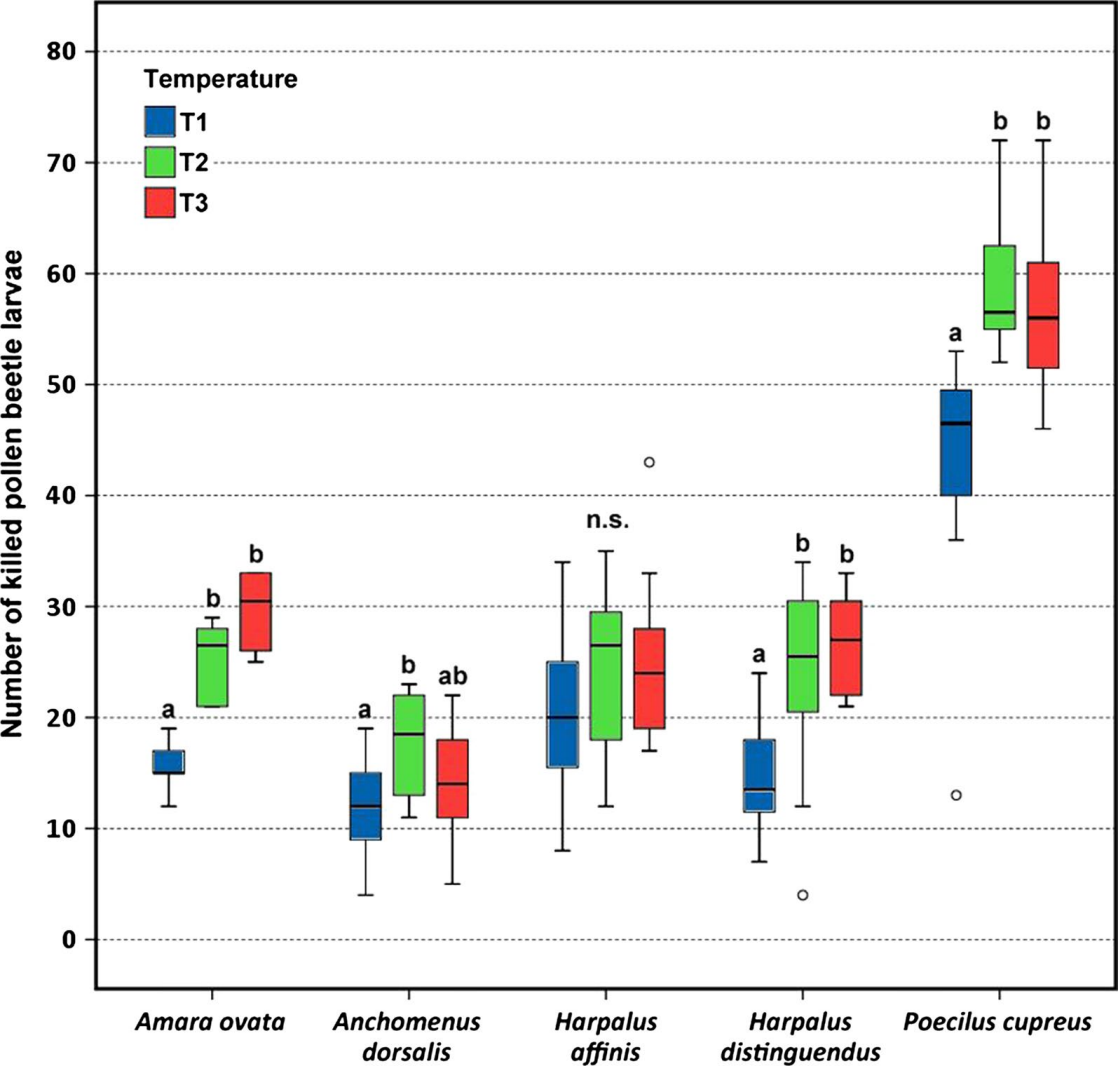
Number of pollen beetle larvae killed by five carabid beetles. Actual temperature (T1) was compared to temperatures increased by 3 °C (T2) and 5 °C (T3). *Box-Whisker plots* show the medians, 25 and 75 % percentiles, 10 and 90 % percentiles, and outlying values outside the percentiles (*open circle*). Different letters denote significant differences among temperatures, *ns* no significant difference (Nemenyi, *P* < 0.05)

**Fig. 2:**
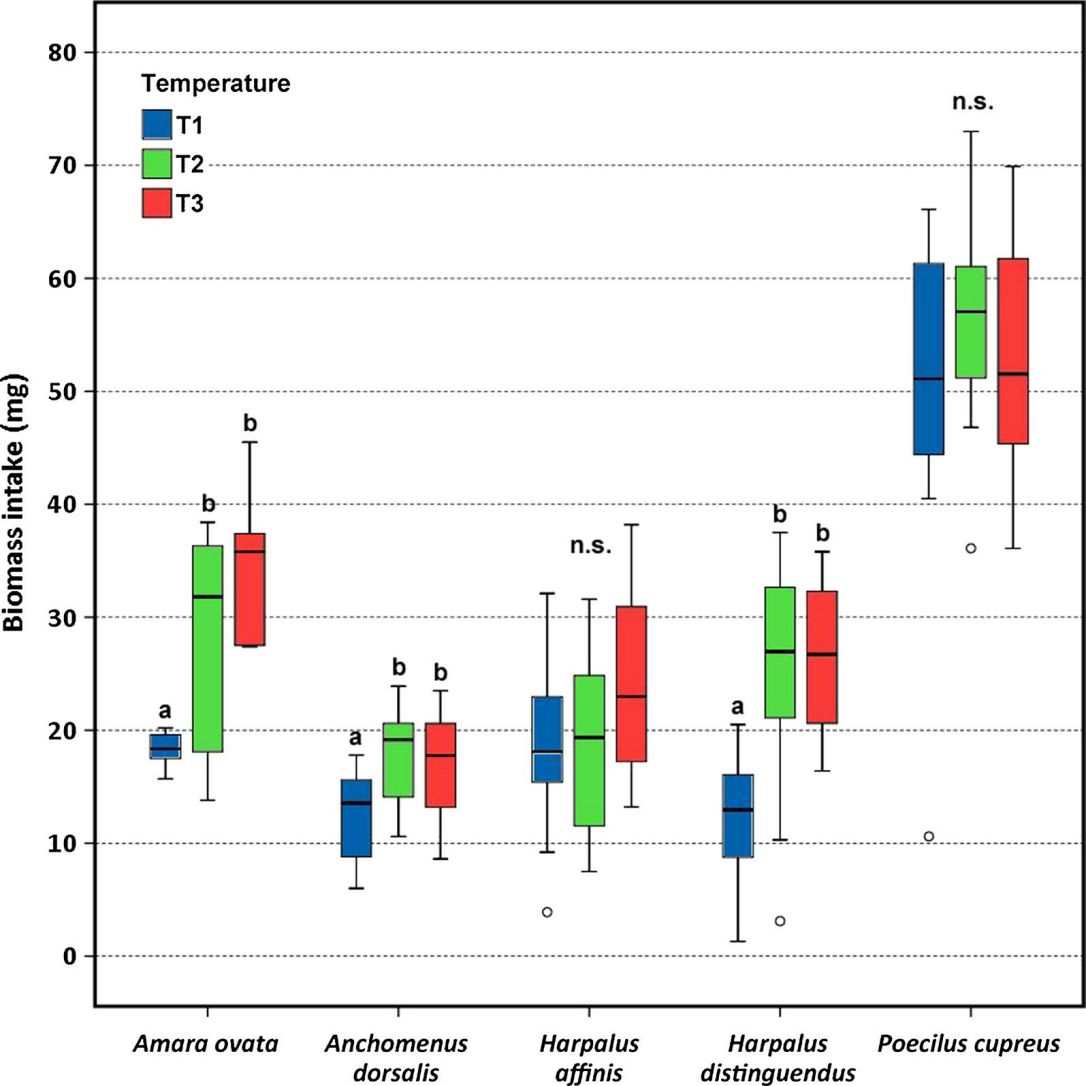
Intake of larval pollen beetle biomass by five carabid beetles. Actual temperature (T1) was compared to temperatures increased by 3 °C (T2) and 5 °C (T3).* Box-Whisker plots* show the medians, 25 and 75 % percentiles, 10 and 90 % percentiles, and outlying values outside the percentiles (*open circle*). Different letters denote significant differences among temperatures, *ns* no significant difference (Tukey,* P* < 0.05)

The factors beetle species and temperature had significant effects on the dependent variables number of killed *Meligethes* larvae and larval biomass ingested by carabids. The interaction term was significant for number of killed *Meligethes* larvae but not for biomass intake (Table 1). Because in the GLM both factors beetle species and temperature were significant, for each single species one-way ANOVAs were performed testing for differences among temperatures relative to the dependent variables. *A. ovata*, *H. distinguendus* and *P. cupreus* killed significantly more *Meligethes* larvae at temperatures 2 (T2) and 3 (T3) compared to temperature 1 (T1). *A. dorsalis* killed significantly more larvae at T2 than T1, and *H. affinis* revealed no significant differences among temperatures (Fig. 1). Biomass intake by *A. ovata*, *H. distinguendus* and *A. dorsalis* was significantly larger at T2 and T3 compared to T1, thus showing similar patterns as for killed larvae concerning the first two species. Biomass intake by *H. affinis* and *P. cupreus* did not significantly differ among temperatures (Fig. 2). Among the five carabids tested *P. cupreus* exhibited the highest values for both number of killed larvae and biomass intake. By considering the varying body mass of the five carabid beetles, we observed concurrent patterns for the number of killed pollen beetle larvae mg^−1^ and intake of larval pollen beetle biomass mg^−1^. *A. dorsalis* killed significantly more larvae mg^−1^ than the other four species, and the same was also true for biomass intake mg^−1^. Killing rate mg^−1^ and biomass intake mg^−1^ by *A. ovata* and *P. cupreus* were significantly larger compared to *H. affinis* and *H. distinguendus* (Table 2).

**Table 1:** GLM showing the influence of carabid beetle species and temperature on the dependent variables number of killed pollen beetle larvae and intake of larval pollen beetle biomass

Dependent variable	Beetle species			Temperature			Beetle species × Temperature		
	D.f.	F	*P*	D.f.	F	*P*	D.f.	F	*P*
Larvae killed	4	222.971	<0.001	2	23.924	<0.001	6	3.194	0.016
Biomass intake	4	146.708	<0.001	2	9.481	<0.001	6	1.837	0.097

The model for killed larvae was analyzed using Poisson distribution

**Table 2:** Number of pollen beetle larvae killed mg^−1^ and intake of larval pollen beetle biomass mg^−1^ by five carabid beetles

Dependent variable (mg^−1^)	*A. ovata*	*A. dorsalis*	*H. affinis*	*H. distinguendus*	*P. cupreus*
Larvae killed	0.950 ± 0.008 a	0.991 ± 0.006 b	0.898 ± 0.005 c	0.898 ± 0.005 c	0.941 ± 0.005 a
Biomass intake	0.894 ± 0.022 a	1.010 ± 0.017 b	0.697 ± 0.016 c	0.713 ± 0.016 c	0.834 ± 0.016 a

Different letters denote significant differences among species (Tukey, *P* < 0.05; mean ± SE)

## Discussion

Four of five carabid species investigated killed more *Meligethes* larvae at rising temperature, and three species ingested more *Meligethes* biomass at higher temperature, thus largely confirming our hypotheses. Thus pollen beetle larvae, being on the way to pupate in the soil, may be killed more by epigeic carabids at higher temperature. This finding sounds promising for biological pest control in regions where climate warming is to be expected.

Our results agree with studies observing that feeding activity and biomass intake by slug feeding carabid beetles increase with rising temperature [10]. Activity of spring breeding carabids, which were tested in the present study, is described to generally increase at higher temperature [24, 25]. This helps to explain the higher killing rate and biomass intake in the present study due to increased energy requirements at T2 and T3, as temperature has long been known to influence metabolism [26]. The metabolic theory of ecology states that higher temperatures lead to higher metabolic rates in ectotherms and this results in higher consumption and digestion. T2 and T3 did never differ significantly. Thus, a further temperature increase (T2 > T3) did not affect carabids additionally. According to existing literature it may be expected that number of killed pollen beetle larvae and biomass intake should significantly increase from T2 to T3. When testing temperatures from 10–28 °C, there was a carabid species whose consumption on seeds increased linearly with increasing temperature, but consumption increase for another carabid was curvilinear and stopped at 20 °C [7]. Such temperature limits might help to explain why a further temperature increase in the present work did not affect carabids additionally.

As *A. dorsalis* and *H. distinguendus*, two of the species that killed more *Meligethes* larvae and ingested more biomass at elevated temperature, are reported as thermophilous [27–30] they appear to be well adapted to higher temperatures. Similarly *A. ovata*, though reported to feed primarily on seeds [31], is obviously also insectivorous as it was observed to kill significantly more larvae and ingest more biomass at T2 and T3. Therefore, from the viewpoint of biological control this species was positively affected by increased temperature. *P. cupreus* killed significantly more *Meligethes* larvae at T2 and T3, and killed much more larvae than the other four species. The highest killing rate is simply because it was the heaviest species tested, which renders it a particularly promising biocontrol agent of *Meligethes* larvae. This argument is substantiated because *P. cupreus* is often observed to be the carabid species with the highest acitvity-density in Central European arable fields [32]. We often observed *P. cupreus* to kill many *Meligethes* larvae without consuming them under our experimental conditions. This may explain why there was no significant difference in biomass intake among the three temperature regimes. Thus, *P. cupreus* did not transfer its increased killing rate at higher temperatures into an increased ingestion of prey biomass. The killing of prey without consuming it, known as "superfluous killing“ or "wasteful killing“, has already been observed for *P. cupreus* when offered different insect prey in the laboratory [33]. *H. affinis* was the only species whose killing rate and biomass intake remained unaffected by temperature. It preferably occurs in dry and sunny open habitats [34–36], which may explain its tolerance towards the different temperature regimes investigated.

There was a negative relationship between predation rates of aphids and the community-average value of body size of ground-dwelling spiders and carabids in cereal fields [37]. Laboratory daily consumption of aphids by predatory beetles considering the predator´s body weight was lowest for large carabids and highest for small staphylinids [38]. Consistently, number of killed pollen beetle larvae and intake of larval pollen beetle biomass related to 1 mg body weight of predators were higher for lightweight carabids compared to heavy ones for four out of five species (body weight: *A. dorsalis* < *A. ovata* < *H. distinguendus* < *H. affinis*). For *H. affinis*, the heaviest of these four beetles, pollen beetle larvae appeared to be a non-attractive prey, which may explain why both the total killing rate and total biomass intake were not affected by increasing temperature. Killing rate and total biomass intake by *P. cupreus* were remarkably high in relation to its body weight. This may be because *P. cupreus* was observed to be particularly active in the Petri dishes, which likely resulted in increased energy requirements.

## Conclusions

Climate simulation models suggest several Central European arable pest insects to increase in number of generations per year [39, 40]. Moreover, *Meligethes viridescens*, which has recently been introduced to Canada, was modelled to increase in abundance for temperature increases between 1 and 7 °C [8], thus covering the temperature increase range of the present study. As climate warming may also be favourable to *M. aeneus* potential consequences of our lab experiment for field population development of *M. aeneus* are not clear so far. Nonetheless our lab results suggest a clear potential for higher feeding of pollen beetle larvae by carabid beetles at rising temperature. As activity-density of carabid beetles in wheat was found to significantly increase when temperature was artificially raised by only 2 °C [9], it can be expected that carabids encounter more pest individuals at soil surface, which in turn most likely increases pest suppression under warmer field conditions. Laboratory studies revealing that carabid beetles feed more at rising temperature span a large range of temperatures of more than 15 °C [7, 10]. The present study is to our knowledge the first one on pest consumption spanning a realistic forecasted climate warming scenario of 3 and 5 °C, thus providing basic knowledge on that neglected research topic.

## Additional file

10.1186/s12898-016-0076-x Raw data on carabid species, temperature, killed pollen beetle larvae and biomass intake.

## Abbreviations

GLM: generalized linear model
T1: actual temperature
T2: temperature increased by 3 °C
T3: temperature increased by 5 °C
ZAMG: Central Institution for Meteorology and Geodynamics Austria
